# QUALITY OF LIFE AND SOCIAL DETERMINANTS PREDICT HOSPICE IN DEMENTIA CAREGIVING DYADS: A MACHINE LEARNING APPROACH

**DOI:** 10.1093/geroni/igac059.2051

**Published:** 2022-12-20

**Authors:** Suzanne Sullivan, Wei Bo, Chin-Shang Li, Wenyao Xu, Yu-Ping Chang

**Affiliations:** University at Buffalo, Buffalo, New York, United States; University at Buffalo, Buffalo, New York, United States; University at Buffalo, Buffalo, New York, United States; University at Buffalo, Buffalo, New York, United States; University at Buffalo, Buffalo, New York, United States

## Abstract

Hospice care is available to assist people with serious illness and their caregivers who wish to age in place, avoid unnecessary hospitalizations, and remain at home through the end-of-life. However, hospice care is under-utilized nationally despite the disproportionate prevalence of end-of-life dementia caregiving burdens among disadvantaged groups. The reasons are unclear, but emerging research suggests that systemic barriers may contribute to underutilization. Commonly used quality-of-life frameworks have long included social determinant of health (SDH) factors such as social, environmental, financial, and healthcare access needs. Investigating the link between quality-of-life and SDH concerns of persons with dementia (PWD) and their caregivers may help identify when a PWD might benefit from hospice care. This study uses machine learning techniques to longitudinally analyze caregiver/care-recipient dyads in the National Health and Aging and Trends Study (NHATS) linked to the National Study of Caregiving (NSOC) (2015-2018) to identify quality-of-life and SDH predictors of hospice use among 117 PWD and their primary caregivers. Results indicate that distinguishing features selected by Information Gain Ratio [IGR] predict that memory rating, receiving food stamps, whether health prevents enjoying life, having trouble chewing or swallowing, diabetes, a regular doctor, and nobody to talk to can predict hospice use well (accuracy=0.6848; sensitivity=0.8244; specificity=0.5371; AUC=0.7425). Quality-of-life/SDH factors are important longitudinal predictors of hospice that can be detected up to three years prior to death. Our study uses inductive, machine learning approaches to provide testable hypotheses for future research to improve the quality of end-of-life care through hospice for PWD and their caregivers.

